# How a Depressive Medical Doctor Profited in the Long-Term from a New and Short Psychological Group-Treatment against Major Depressive Disorder

**DOI:** 10.3390/ijerph18041925

**Published:** 2021-02-17

**Authors:** Daryl Wayne Niedermoser, Nadeem Kalak, Martin Meyer, Nina Schweinfurth, Marc Walter, Undine E. Lang

**Affiliations:** 1 Department of Addictive Disorders, Psychiatric University Clinic Basel, 4002 Basel, Switzerland; nadeem.kalak@upk.ch (N.K.); MartinPhilipp.Meyer@upk.ch (M.M.); nina.schweinfurth@upk.ch (N.S.); marc.walter@upk.ch (M.W.); Undine.Lang@upk.ch (U.E.L.); 2 Department of Economics, Kalaidos University of Applied Sciences, 8050 Zürich, Switzerland

**Keywords:** workplace-related stress, interpersonal psychotherapy, depression, ability to work, follow-up

## Abstract

*Background:* Individuals suffering from major depressive disorder (MDD) often describe workplace-related stress as one of the main causes of their disorder. Here, we present the story of a 33 year old “Bob” (a pseudonym) who suffered from a moderate (Hamilton-21 = 18) major depressive episode. Workplace-related stress seemed to be the main stressor for Bob at the time. We were interested in long-lasting effects of a newly established group called “work-related interpersonal Psychotherapy, W-IPT”. W-IPT consists of eight weekly 90 min sessions. The follow-ups were 12 weeks after the group-treatment and 18 months later. Bob was chosen because he agreed in advance to participate in a follow-up. We were interested if the group-treatment of W-IPT also has a persistent positive effect. *Case presentation:* We present the case of a 33-year-old man “Bob”. He was included in our previous published pilot-study 2020 with diagnosed moderate MDD, and he attended the group treatment. This case report focuses on a follow-up period of 18 months. A structured clinical interview for DSM-IV was carried out in order to be included in the study, and no comorbid diagnoses were detected. *Conclusions:* However, the psychotherapeutic effects in this case seem enduring and prolonged. Of course, additional research to study the long-term effects of W-IPT is needed, and more patients need to be included.

## 1. Introduction

Major depressive disorder (MDD) is a common and debilitating mental disorder. MDD is associated with an increase in disability [1]. Gold-standard treatments of MDD from moderate to severe consist mainly in treatment with antidepressants, i.e., serotonin-reuptake inhibitors (SSRIs) and psychotherapy guidelines (German Association of the Scientific Medical Societies (AWMF), National Institute of Clinical Excellence (NICE)). 

Recent reviews and meta-analyses of antidepressants have again raised the question of efficacy (e.g., [2,3]. Munkholm et al. 2019 [2] concluded that “the evidence does not support definitive conclusions regarding the benefits of antidepressants for depression in adults” (p. 1), and Hengartner [3] wrote in a commentary title that there were some “misleading arguments about the efficacy of antidepressants”.

However, a very important component of antidepressants is their unpleasant side effects, including nausea, weight gain, fatigue, insomnia, and shakiness. However, when patients are trying to reduce or to stop the intake of antidepressants, withdrawal symptoms, similar to those of benzodiazepines [4,5] and antipsychotics [6], can be noticed. As early as 2003, the World Health Organization (WHO) pointed out that there have been withdrawal syndromes reported around the world for SSRIs, and health problems in which a sufficient number of criteria for real addiction have been met [7]. This can cause fear among patients and can easily be misinterpreted (by patients and experts) as a relapse or a recurrence of the initial illness [6]. Most of the measureable unpleasant side effects were significantly increased when compared with placebo treatment [8]. This intolerance could be one of the reasons why people with MDD often stop taking antidepressant medication [3,4,9,10,11]. Other evidence-based treatment options include neuromodulation [11,12,13], cognitive-behavioral interventions [14,15], or regular physical activity [16,17,18,19].

Patients with MDD often report workplace-related stress [20]. Tension at work, perceived low job control and support, high psychological demands, and insecurity in the workplace have all been confirmed as predictors of depression [20,21,22,23,24,25].

Recent studies [26,27] show that people with MDD are at higher risk of losing their current job compared to healthy people [26,27,28] and to have greater difficulty getting back to and staying in their jobs when they return to work after a period of illness-related unemployment [26,27,28].

Specific treatments to reduce workplace-related stress in people with MDD might reduce economic costs to the public and individual [27,28,29,30,31].

The newly developed workplace-related interpersonal psychotherapy (W-IPT) in groups is a specific treatment to focus on workplace-related issues in individuals with MDD [32,33].

Originally, IPT was developed as a brief psychotherapeutic treatment aimed at treating symptoms of acute depressive disorder and interpersonal problems. In numerous trials, the effectiveness of the treatment of MDD has been demonstrated (cf. meta-analysis from: Cuijpers, Van Straten [34]). The W-IPT focuses on problems that arise in the workplace [32], such as social or interpersonal, bullying, transition, burn- or boreout, effort-reward-imbalance, and work–life imbalance (see Table 1).

The main focus of the group program was not only on theoretical input but also on practicality. For example, cognition and its interpretation were reflected in the group, along with the importance of a balance between work and leisure, and the potentially problematic effects of a perceived imbalance between demands and control. Overall, the focus was on four areas: work–life balance, demanding control, valuing work, and returning to work (see Table 1).

Unfortunately, there were not many long-term evaluations concerning workplace-related interpersonal psychotherapy. That is why we decided to follow up on a study participant. This case report aims to shed light on possible long-term effects of the W-IPT treatment of MDD. Specialists often suffer from MDD as well. This case covers two important areas, and we therefore believe it is of special interest.

## 2. Case Description

The following case is a 33-year-old male with no psychological or psychiatric history. In his youth, he probably showed signs of a social phobia, but was remitted when he started the study. For future references, we are calling him “Bob”. Bob grew up in a typical family of immigrants in a rural city in the Canton of Basel-Country (Switzerland). He was the only child in his family, and they lived in a small flat. At the age of seven years, his father died aged 44 of lung cancer. This traumatic incident had a major impact on his relationship with his mother and was probably the reason why he chose to study human medicine. He was an excellent pupil and student and a quick learner.

At his workplace, a well-known public hospital in Switzerland, he realized that he was stressed. He had ruminations, could not sleep well, and was more anxious than before. Therefore, he concluded that most probably he was having a mental problem, i.e., a depressive episode. He realized that something needed to change, and that is why took part in our W-IPT study [33].

**Figure 1:**
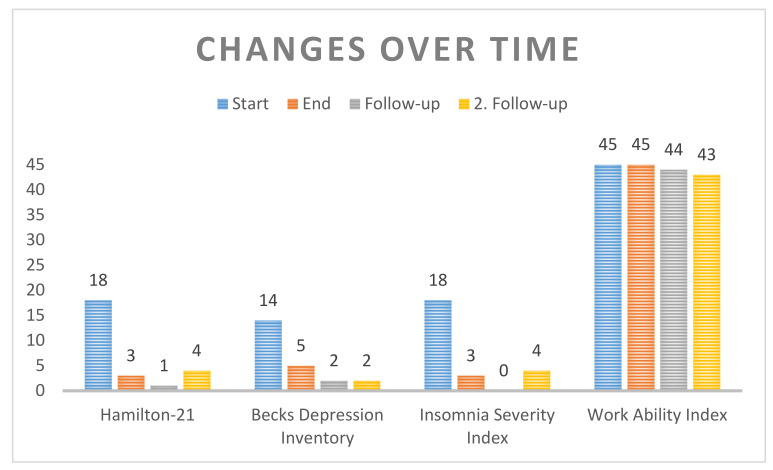
Changes over time.

The applied W-IPT for 8 weeks was successful. Bob was able to adapt. According to him, he profited most from the communication of the Kiesler Cirumplex model [33,35]. Moreover, it helped him to be aware of his self-awareness and self-care. In addition, this whole exchange during the study (especially initial contact) helped him to change his originally negative attitude towards psychiatry and psychology. He realized that he could engage in daily habits to improve his situation and support his health (by changing his communication habits and doing physical activity on a regular basis). The change in his attitude during our W-IPT group sessions seemed to be essential in Bob’s recovery process. He attributed it to his understanding of the Kiesler Circumplex model and its relationship to his work environment. In addition, he developed an intrinsic motivation for further understanding his interpersonal relation style. After that, his sleep onset insomnia disappeared and he reported improved recovery during nighttime sleep. This was another motivational factor in his own social life. Twelve weeks after the study ended (with no additional therapy), he showed no signs of clinically relevant depression (Hamilton-21 = 1) (see Table 2 and Figure 1). Furthermore, he reported that he was eager to learn more about himself and to tackle some older issues from his past. Therefore, he decided to get some additional professional help. He went to a psychiatrist and a psychotherapist. The psychiatrist was educated in schema therapy (Jeffrey Young) and helped him to tackle his childhood trauma. In addition, as a supportive treatment he took bupropion (Wellbutrin) for six months. After that, he went to a psychotherapist for stabilization as a preventive and supportive strategy. During all that, he finalized his doctoral degree and transferred to another institute in Switzerland. Today, he is doing very well without being in psychological or psychopharmacological treatment (Hamilton-21 = 4) (see Table 3). According to him, he often thinks about the group treatment, which motivated him to start to tackling his past and to overcome his inhibition and begin to change. He recommends the treatment to anybody who wants to reduce workplace-related stress.

## 3. Discussion

Bob took part in an eight-session group program, designed to focus on workplace-related stress. Subjectively and objectively, it was a significant success. He was intrinsically motivated to face his problems. The follow-up from Bob 18 months after he participated was quite impressive. He has another job (he still works as a medical doctor) and he is doing “fine”. This group-based treatment for quite a short period seems to have initiated a long-lasting stable and positive effect. Although Bob was getting additional help from a psychiatrist and medication, the group treatment helped and motivated him to keep this change alive and long lasting. A spark was ignited. We believe this huge effect occurred within the first 12 weeks, particularly as a positive factor from our group-based W-IPT treatment.

Bob was not a complex case, as mentioned before, but he exhibited quite an astonishing treatment effect within the first weeks. Furthermore, we believe that Bob was able to transfer the effect with additional help into a stable effect over time (measured in self- and expert ratings). Importantly, although he was initially not taking any antidepressants or psychotherapeutic treatment, he changed over the course of 18 months. Other possible confounders except the positive group experience may be his graduation from doctoral studies (MD PhD); therefore, his new motivation and new job should be mentioned here as well. Three major benefits of this W-IPT training shall be highlighted: first, it is an effective intervention that can be implemented in any company; second, it is short and therefore not an expensive treatment; and third, it seems to have a stable effect over time (an 18-month period).

## 4. Conclusions

The W-IPT group program should be considered for treating patients with workplace-related stress and mild-to-moderate MDD. There are also many questions about the efficacy of antidepressants, as mentioned in the introduction [2,3,9,10,36].

We believe that these results might be of broader interest to experts. Short- and long-term costs for employers as well as for employees could be reduced. For example, the cost of being less productive or of absent employees lead to several high costs per employee [27,29,31,37]. Public health costs should also be mentioned here. Last, this program is easy and quickly learned (no absence from work is needed). It even can be implemented in the workplace, which could be quite beneficial for companies. Short and efficient treatment in groups seems to be easy to implement and is especially economical. Possible uses include (1) companies with modern occupational health management, and (2) individuals experiencing workplace-related stress for secondary or tertiary prevention.

## Figures and Tables

**Table 1:** Details of the four workplace-related interpersonal psychotherapy (W-IPT) modules.

Module	Goals
1 Work–life balance	Analysis of the work situation (risk factors, resources) Introduction of being at workplace and self-care (energy givers) Asking for help, social support at work
2 Demanding control	Facing interpersonal conflicts and difficult changes at work Communication at work (limit-setting) Addressing effort–reward imbalances
3 Valuing work	Identifying personal values (increasing work–life balance) Applying communication skills at work
4 Returning to work	Association of (work-)stress and depressive symptoms Summary of balance (goals) Return-to-work attitude

**Table 2:** Overview over time of numbers.

	Start	End	Follow-Up	2. Follow-Up (15 Months)
Hamilton-21	18	3	1	4
Becks Depression Inventory	14	5	2	2
Insomnia Severity Index	18	3	0	4
Work Ability Index	45	45	44	43

**Table 3:** Overview over time of interpretations.

	Start	End	Follow-Up	2. Follow-Up (15 Months)
Hamilton-21	Moderate depression	No depression	No depression	No depression
Becks Depression Inventory	Mild depression	No depression	No depression	No depression
Insomnia Severity Index	Clinical insomnia, moderate severity	No clinically significant insomnia	No clinically significant insomnia	No clinically significant insomnia
Work Ability Index	Very good work ability	Very good work ability	Very good work ability	Good work ability

## Data Availability

All data underlying the results are available as part of the article and no additional source data are required.
